# Genetic divergence and one‐way gene flow influence contemporary evolution and ecology of a partially migratory fish

**DOI:** 10.1111/eva.13712

**Published:** 2024-06-21

**Authors:** Katie M. Kobayashi, Rosealea M. Bond, Kerry Reid, J. Carlos Garza, Joseph D. Kiernan, Eric P. Palkovacs

**Affiliations:** ^1^ Department of Ecology and Evolutionary Biology University of California Santa Cruz California USA; ^2^ Fisheries Collaborative Program, Institute of Marine Sciences University of California Santa Cruz California USA; ^3^ Southwest Fisheries Science Center National Marine Fisheries Service Santa Cruz California USA; ^4^ Area of Ecology and Biodiversity, School of Biological Sciences University of Hong Kong Hong Kong Hong Kong, SAR; ^5^ Department of Ocean Sciences University of California Santa Cruz California USA

**Keywords:** anadromy, contemporary evolution, eco‐evolutionary dynamics, gene flow, intraspecific variation, *Omy05*, *Oncorhynchus mykiss*, rainbow trout, residency, secondary contact, steelhead

## Abstract

Recent work has revealed the importance of contemporary evolution in shaping ecological outcomes. In particular, rapid evolutionary divergence between populations has been shown to impact the ecology of populations, communities, and ecosystems. While studies have focused largely on the role of adaptive divergence in generating ecologically important variation among populations, much less is known about the role of gene flow in shaping ecological outcomes. After divergence, populations may continue to interact through gene flow, which may influence evolutionary and ecological processes. Here, we investigate the role of gene flow in shaping the contemporary evolution and ecology of recently diverged populations of anadromous steelhead and resident rainbow trout (*Oncorhynchus mykiss*). Results show that resident rainbow trout introduced above waterfalls have diverged evolutionarily from downstream anadromous steelhead, which were the source of introductions. However, the movement of fish from above to below the waterfalls has facilitated gene flow, which has reshaped genetic and phenotypic variation in the anadromous source population. In particular, gene flow has led to an increased frequency of residency, which in turn has altered population density, size structure, and sex ratio. This result establishes gene flow as a contemporary evolutionary process that can have important ecological outcomes. From a management perspective, anadromous steelhead are generally regarded as a higher conservation priority than resident rainbow trout, even when found within the same watershed. Our results show that anadromous and resident *O. mykiss* populations may be connected via gene flow, with important ecological consequences. Such eco‐evolutionary processes should be considered when managing recently diverged populations connected by gene flow.

## 1 INTRODUCTION

In recent decades, it has become well recognized that evolutionary processes occur on time scales that have important implications for different levels of ecological organization (e.g., populations, communities, ecosystems) (Hendry, 2016; Pelletier et al., 2009). For example, if adaptive evolution drives variation in ecologically relevant traits/species (e.g., physiology, morphology, behavior, life history), it is possible for such intraspecific variation to elicit cascading population, community, and ecosystem effects (Bolnick et al., 2011; Des Roches et al., 2018; Hendry, 2016). While extensive research has focused on how isolated populations diverge evolutionarily, and sometimes ecologically, from their founding populations (Bassar et al., 2010; Harmon et al., 2009; Post et al., 2008), considerably less attention has been given to the subsequent potential for newly adapted populations to have evolutionary and ecological effects on their founding population. One way this can happen is through gene flow, if divergent populations maintain connectivity through migration or dispersal (Farkas et al., 2015). By studying the effects of gene flow between divergent and founding populations, we expand our understanding of the eco‐evolutionary consequences of intraspecific variation.

Empirical evidence documenting the eco‐evolutionary consequences of gene flow remains highly limited and context dependent. Gene flow is commonly thought to constrain genetic and phenotypic divergence of populations inhabiting different environments through homogenization (e.g., Haldane, 1930; Hendry & Taylor, 2004; Muhlfeld et al., 2009; Nosil & Crespi, 2004). However, there is likewise evidence that local adaptation can be maintained and even reinforced in the presence of gene flow, since gene flow can provide beneficial alleles for selection to act on (Fitzpatrick et al., 2015, 2020). In either case, gene flow can influence observed genetic and phenotypic variation, thus creating opportunities for eco‐evolutionary responses (Farkas et al., 2013, 2015; Garant et al., 2007; Miller et al., 2019). Gene flow may be of particular importance for mediating the spread of keystone genes, which can elicit strong community or ecosystem responses through effects on ecologically important phenotypes (Nosil & Gompert, 2022; Skovmand et al., 2018).

In riverine ecosystems, population fragmentation by natural and anthropogenic barriers such as waterfalls and dams has been shown to disrupt gene flow from downstream to upstream, reduce genetic diversity upstream, and drive adaptation to new environments (Zarri et al., 2022). Instream barriers often exhibit asymmetry in their permeability—permitting downstream dispersal and gene flow while limiting or preventing upstream dispersal (Junker et al., 2012; Kelson, Miller, et al., 2020; Peacock et al., 2016; Raeymaekers et al., 2009). Previous studies have shown that unidirectional gene flow originating from divergent populations above barriers can contribute to increased genetic diversity in populations below barriers (Crispo et al., 2006; Hänfling & Weetman, 2006; Harris et al., 2015; Junge et al., 2014; Reis et al., 2015). Similarly, studies of secondary contact in migratory fishes have shown that restoring gene flow between populations adapted to different environments can influence life‐history expression and population dynamics (Reid et al., 2020). However, evolutionary outcomes for recipient populations can vary greatly due to system‐specific factors such as dispersal rates, reproductive barriers, and environmental gradients (Farkas et al., 2015; Fitzpatrick et al., 2019; Garant et al., 2007; Labonne & Hendry, 2010). As such, the long‐term phenotypic and ecological consequences of asymmetrical gene flow remain unclear.

The salmonid species *Oncorhynchus mykiss* (Walbaum, 1792) provides an ideal opportunity to study the eco‐evolutionary effects of one‐way gene flow between divergent populations. *O. mykiss* exhibit an impressive range of life‐history strategies, which are often dichotomized into two primary ecotypes. The common name ‘steelhead’ is applied to members of the species that exhibit an anadromous life history; whereby they are born in freshwater, migrate to the ocean as juveniles, and then return to freshwater to spawn (Kendall et al., 2015; Quinn, 2018). Conversely, the name ‘rainbow trout’ refers to conspecifics that remain in freshwater for their entire life cycle (Kendall et al., 2015; Quinn, 2018). *O. mykiss* populations are well documented to undergo rapid adaptive evolution in response to environmental change from both a genetic and phenotypic perspective (Kendall et al., 2015; Pearse et al., 2014; Sloat et al., 2014). In many locations, anadromous steelhead have been introduced above barriers to migration, such as waterfalls and dams, which impede their ability to return to above barrier habitat and reproduce if they outmigrate (Martínez et al., 2011; Pearse et al., 2009; Willoughby et al., 2018). The resulting selection against downstream migration has been documented to drive the parallel evolution of resident life histories (i.e., rainbow trout) across much of their historic range (Hayes et al., 2012; Pearse et al., 2009; Phillis et al., 2016). In populations from coastal California (USA), migratory life‐history strategy has been associated with a large autosomal inversion on chromosome Omy05, such that individuals with the homozygous ancestral (AA) arrangement were more likely to migrate compared to those with homozygous rearranged (RR) genotypes (Kelson et al., 2019; Martínez et al., 2011; Pearse et al., 2014, 2019). Such a close association between genotype and phenotype on an individual level provides a unique opportunity to reconcile evolutionary histories revealed by genetic analysis with the ecological effects driven by their associated phenotypes.

In some river systems, individuals from resident *O. mykiss* populations above barriers may descend downstream (either volitionally or via displacement during high stream flows), thus creating the potential for one‐way gene flow from resident‐adapted ‘rainbow trout’ populations to their anadromous ‘steelhead’ ancestors below. However, empirical evidence documenting such dispersal remains limited (Bowersox et al., 2016; Hayes et al., 2012; Pearse et al., 2009). It is well established that resident and anadromous ecotypes can reproduce with one another in sympatry (Avise et al., 2002; Kendall et al., 2015; Seamons et al., 2004; Shapovalov & Taft, 1954), but some studies have suggested mechanisms that would maintain reproductive isolation such as timing of reproduction, mate choice preferences, or reduced hybrid fitness (Hendry et al., 2000; Kirkpatrick, 2001; McMillan et al., 2007; Pearse et al., 2009; Zimmerman & Reeves, 2000). In either case, if residents occasionally descend barriers and successfully reproduce, below‐barrier populations could exhibit an increased frequency of resident genotypes and a corresponding decrease in their phenotypic propensity to migrate (Hayes et al., 2012).

Variation in migratory strategy may have substantial implications for the density and size structure of *O. mykiss* populations and, by extension, the management and ecology of freshwater ecosystems. Migration to the marine environment and/or highly productive estuarine rearing habitat typically allows individuals to achieve larger body sizes at maturation compared to those that remain in freshwater (Bond et al., 2022; Hayes et al., 2008; Kendall et al., 2015). Given the positive relationship between body size and fecundity in *O. mykiss* and other salmonids (Quinn, 2018), the reproductive potential of resident and migrant ecotypes can differ by orders or magnitude (Hayes et al., 2008, 2012). However, resident rainbow trout typically experience higher rates of survival and iteroparity (Fleming & Reynolds, 2004). As a result, in stream regions that predominantly contain migratory genotypes, population size structure is characterized by high densities of young‐of‐year (age‐0+) fish. Conversely, stream regions predominantly containing resident genotypes are characterized by more complex size structures, due to higher densities of older (age‐1+ and age‐2+) fish (Kelson, Miller, et al., 2020). Thus, where gene flow leads to increased genotypic variation, it may be possible for combinations of anadromous offspring and older residents to drive increasingly complex population structures. Changes in density and size structure of the population may, in turn, have important implications for a number of ecological processes—from growth and survival, to trophic control and ecosystem function (Grossman & Simon, 2020; Kelson, Miller, et al., 2020; Milner et al., 2003; Moore, 2006). However, current management and conservation strategies typically treat anadromous steelhead and resident rainbow trout separately, including many instances in which conservation protections exclude adjacent populations based on life‐history type. Thus, understanding how these ecotypes interact and affect each other is of critical importance for designing effective management strategies.

Here, we use a combination of molecular and field‐based approaches to consider the eco‐evolutionary consequences of an introduction of *O. mykiss* above waterfalls on two tributaries, which prevent upstream passage but enable downstream gene flow back into the ancestral founding population. First, we use SNP genotyping and mark–recapture data to examine how populations evolve where there is potential for downstream gene flow. Then, we use field survey data to explore how resultant life‐history variation influences the population ecology (density, size structure, sex ratio) in *O. mykiss*. We address the following specific questions: (Q1) Is there genetic evidence of downstream dispersal from resident‐adapted fish above migration barriers to their anadromous founding population below? (Q2) Is there phenotypic evidence of downstream dispersal from resident‐adapted fish above migration barriers to their anadromous founding population below? (Q3) How does the resulting variation in life history strategy influence the density, size structure, and sex ratio of populations? Our study provides insight about how gene flow from recently diverged populations may impact the evolution and ecology of the founding populations.

## 2 METHODS

Here, we used a historical translocation of anadromous *O. mykiss* above waterfalls on two tributaries of a coastal California watershed as the experimental basis for studying the effects of genetic divergence and one‐way gene flow on a founding population. We integrated historical records and paired surveys above and below barriers on two tributaries to explore how variation in downstream dispersal and gene flow influence the distribution of genotypes, phenotypes, and population density and size structure. We sampled *O. mykiss* populations at a number of study sites distributed across the watershed. In order to determine whether gene flow from resident to anadromous populations has occurred (Q1), we used single nucleotide polymorphism (SNP) data at both neutral and adaptive loci to analyze patterns of population differentiation and inferred ancestry. Then, we asked whether phenotypic variation reflected patterns of one‐way dispersal from above to below waterfalls (Q2) using mark–recapture data from genotyped fish. Finally, we explored the consequences of one‐way dispersal for population ecology (Q3) using depletion survey data and genetic *Omy05* and sex assignments to estimate and compare density, size structure, and sex ratio among our populations. All data analyses were performed in *R* v4.3.1 (R Core Team, 2013) unless otherwise noted.

### 2.1 Study system

**FIGURE 1:**
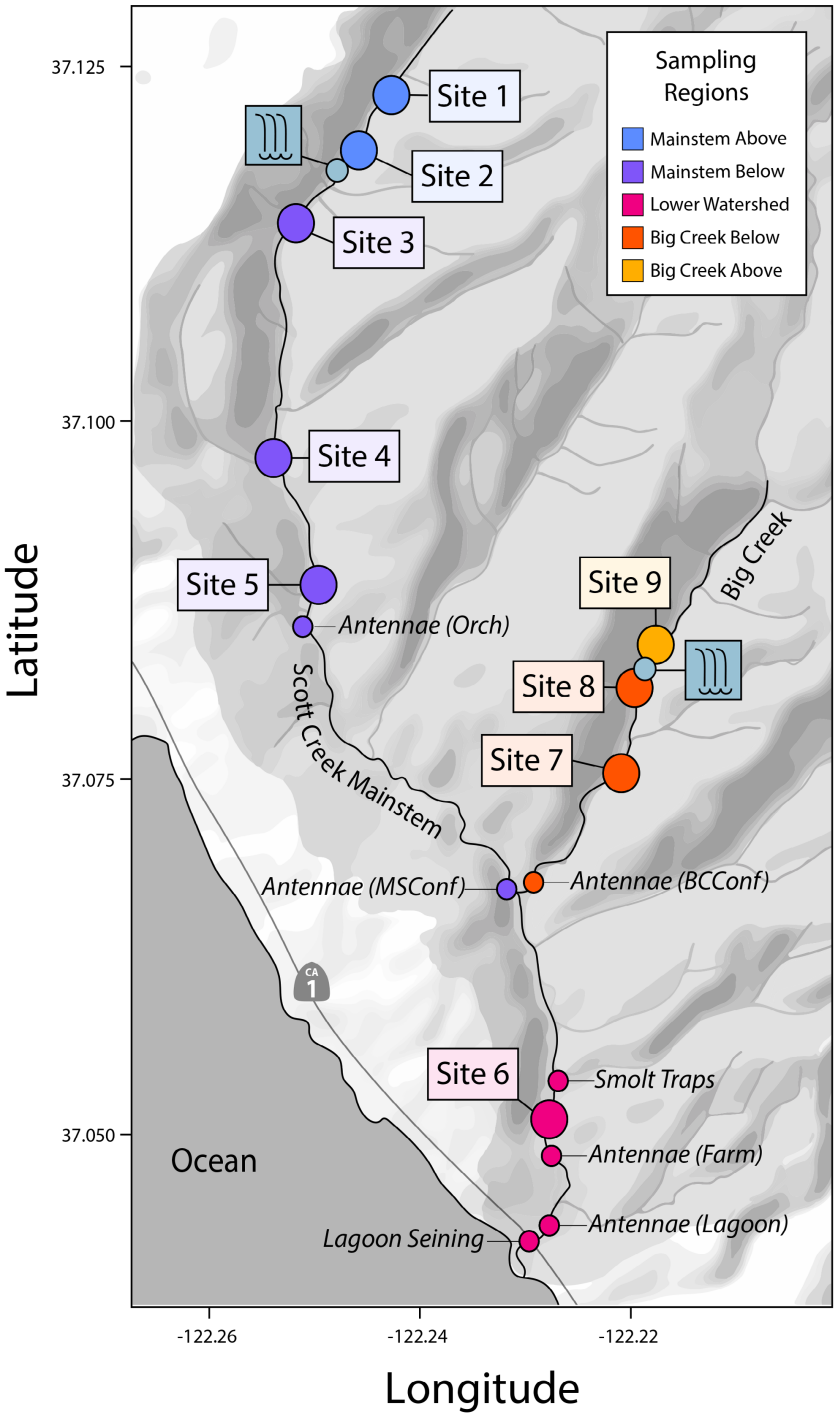
The Scott Creek (Santa Cruz County, California, USA) watershed served as the experimental landscape for our study. Study sites (large circles) were distributed among five sampling regions separated by two waterfall barriers (teal squares) and a major stream confluence. Life‐cycle monitoring sites (small circles) served as additional encounter opportunities for tracking migratory behavior.

Scott Creek is a small (~70 km^2^), coastal watershed, located ~100 km south of San Francisco in Santa Cruz County, CA (Figure 1). Scott Creek is the site of a life‐cycle monitoring station (Adams et al., 2011; Hayes et al., 2012), which provides quantitative information on all life stages of two anadromous salmonid species in the basin–coho salmon (*O. kisutch*) and steelhead trout (anadromous *O. mykiss*). The mainstem of Scott Creek (hereafter ‘Mainstem’) and its largest tributary (Big Creek) provide the majority of ~23 km of spawning and rearing habitat for anadromous salmonids in the watershed. Natural waterfalls on both the Mainstem (a ~10 m, high gradient cascading waterfall) and Big Creek (a ~35 m vertical waterfall) serve as barriers to migration, separating the anadromous portion of the watershed from as much as ~12 km and ~21 km of upstream resident habitat on the Mainstem and Big Creek, respectively. Historical records indicate that *O. mykiss* from the anadromous below‐barrier population were transplanted at least once above both falls in 1910, and have since established resident populations above both falls (Pearse et al., 2009, 2014). Previous work in the watershed has demonstrated that all three populations originate from a shared founding population that was almost entirely anadromous (Shapovalov & Taft, 1954). Strong selection against outmigration from over the falls has resulted in genetically differentiated resident populations in less than 100 years (Hayes et al., 2004; Pearse et al., 2014; Phillis et al., 2016). Despite strong evidence for rapid parallel evolution of residency above both falls, individuals from one of these above‐barrier populations (Big Creek) have been previously documented descending Big Creek Falls (Pearse et al., 2009). Given the common origin, spatial replication, and potential for at least one case of downstream dispersal, the Scott Creek system provides a unique opportunity to study the consequences of gene flow from divergent populations.

In 2017, we identified nine 100‐m stream reaches to serve as long‐term monitoring sites within the Scott Creek watershed (Figure 1). The sites were distributed across the Mainstem (*N* = 6) and Big Creek (*N* = 3) and included locations above and below barriers to anadromous migration. Site selection was non‐random and based on expected life‐history variability and sampling considerations (e.g., site accessibility). Additionally, we sought to maximize the distance between sites so that we could reliably assume that individuals were not moving between sites during annual sample periods.

### 2.2 Data collection

#### 2.2.1 Field data collection

We sampled *O. mykiss* populations at each site annually across three consecutive years (2017–2019). Sampling took place within a 2‐week window during low (base) flow conditions (August/September) to minimize the potential for individuals to disperse among sampling sites. Environmental and hydrological conditions remained fairly constant throughout each annual sampling period. During each fish sampling event, we installed block nets (6 mm mesh) at the upstream and downstream ends of the site and collected fish from the area between the nets using a backpack electrofisher (Model LR‐24; Smith‐Root Inc., Vancouver, WA, USA). To quantify fish abundance and size distribution at each site, we employed multiple‐pass depletion (removal) methods, completing three passes of equal effort by time in most cases. However, additional passes were completed when cumulative catch increased by more than 50% between the previous two passes.

Following capture, we anesthetized *O. mykiss* with tricaine methanesulfonate (MS‐222; Western Chemical Inc., Ferndale, WA, USA), measured for fork length (FL; ± 1.0 mm) and wet mass (± 0.1 g), and excised (clipped) a small (~0.5 cm^2^) portion of the upper caudal fin for genetic analysis and sex determination. To assess fish growth and movement, all captured individuals ≥65 mm FL were issued a 12‐mm passive integrated transponder (PIT) tag (Oregon RFID Inc., Portland, OR, USA) via intraperitoneal injection.

We used mark–recapture sample methods, including a mixture of physical capture and PIT tag antenna detection data, to monitor fish movement after tagging (Figure 1). The initial marking of individuals occurred at each of the nine study sites and recapture information was generated year‐round through a variety of life cycle monitoring efforts, including passive detection events at two stationary PIT tag antenna arrays (*N* = 3404); electrofishing surveys (*N* = 654), estuary/lagoon seining (*N* = 54), and downstream migrant trapping (*N* = 54). We recorded the geographic location of each observation, and for physical recaptures, we re‐measured the individual for FL and mass. Additionally, we used data generated at two stationary PIT tag antenna arrays to infer the emigration of individuals out of the watershed.

The capture and handling of ESA‐listed *O. mykiss* was authorized by the National Marine Fisheries Service under Section 10(a)(1)(A) permit No. 17292‐2A. Fish handling procedures were carried out in accordance with approved protocols from the Institutional Animal Care and Use Committee at the University of California, Santa Cruz (Protocol No. KIERJ1604_A1).

#### 2.2.2 Laboratory data collection

Caudal fin tissue samples were extracted in 96‐well plates using the DNeasy Blood and Tissue Kit following the manufacturer's specifications with the BioRobot 3000 (Qiagen Inc., Gaithersburg, MD, USA). Individuals were genotyped using a 95‐SNP panel developed for performing genetic stock identification and parentage‐based analysis in *O. mykiss*, following the methods of Abadía‐Cardoso et al. (2013). Two negative controls were included in each array, and genotypes were called using SNP Genotyping Analysis Software (Fluidigm, South San Francisco, CA, USA). Additionally, a Y chromosome‐linked sex identification assay was used to categorize individuals as male or female (Brunelli et al., 2008).

### 2.3 Data analysis

#### 2.3.1 Genomic relationships and signatures of gene flow

We analyzed SNP data at neutral loci to identify patterns of population differentiation and ancestry to test for gene flow. We used the Microsatellite Toolkit v3.1 software program to review alleles and basic statistics for each sampling location prior to performing any subsequent analyses (Park, 2001). Out of 2367 genotyped samples, we removed 14 individuals who were missing genotype calls at 10 or more loci (i.e., >10% missing SNP data). We retained 92 of 95 loci for population and family analyses, removing two loci (*SH114448.87* and *Omy.R04944*) that map to the *Omy05* inversion and are subject to selection, and one locus (*SH127645.308*) that was fixed in the population. To account for fish that were resampled in multiple years, we screened for pairs of samples that differed at a maximum of two alleles (i.e., >98% matching), and retained only one sample from each duplicate pair (*N* = 95) selected at random for population and family analyses. To account for the potential effects of family structure on population estimates, we identified groups of full siblings in our dataset and excluded all but one full sibling from each group following the protocol outlined by Garza et al. (2014). We used the program *COLONY* to identify full siblings, using a full likelihood estimation model (Jones & Wang, 2010). We filtered for families of full siblings (*N* = 1488) with inclusive probability greater than or equal to 95% (i.e., Prob(Inc.) ≥ 0.95), meaning that there was a high probability the sibling group accurately represented members of a single family (Figure S1). We selected one individual at random from each full‐sibling group larger than two (*N* = 136; Table S1) to include in our final dataset for population genetic analyses.

The software program *GenePop* was used to calculate observed versus expected heterozygosity for each SNP and estimate values of *F*_ST_ between all site and year pairs (Rousset, 2008). We then used the program *STRUCTURE v 2.3* to identify the ancestry of individuals and explore potential patterns in gene flow (Pritchard et al., 2000). *STRUCTURE* uses cluster analysis to assign proportional ancestries to individuals based on locus‐specific allele frequencies. We repeated five runs for each assumed number of clusters (*k*) from *k* = 1 through *k* = 6 (10^4^ burn‐in period, 10^4^ reps), and selected a final value for *k* that maximized the structure present in the data while still considered biologically reasonable to test our hypothesis (Figure S2; Porras‐Hurtado et al., 2013). We summarized gene flow patterns from our ancestry data in two ways. First, we used the proportional ancestry assignments (*Q* values) computed by *STRUCTURE* to quantify the number of individuals who were assigned majority ancestry to a given cluster (*Q* > 0.5), relative to their capture location in the watershed. We also calculated the total fraction of ancestry from each cluster (i.e., the sum of *Q* values multiplied by the number of individuals) at each site.

We used the two loci from our SNP panel located within the *Omy05* inversion to identify ancestral (A) and rearranged (R) haplotypes and categorized individuals as having AA, AR, or RR genotypes at *Omy05* (Pearse et al., 2014, 2019). Exact tests for Hardy–Weinberg equilibrium at *Omy05* were conducted using the *HardyWeinberg* package in *R* (Graffelman, 2015; R Core Team, 2013). For each study site, we calculated the relative frequency of each *Omy05* genotype as the number of individuals assigned that genotype divided by the total number of fish. We predicted that the proportion of R alleles and RR genotypes would increase where upstream ancestry was greater, including populations above barriers, as well as sites below the falls where we observed higher rates of above‐barrier ancestry due to gene flow.

#### 2.3.2 Physical movement and migratory behavior

To explore phenotypic evidence for downstream dispersal, we analyzed mark–recapture data from life‐cycle monitoring surveys following the methods outlined in Pearse et al. (2019). Our initial data set consisted of fish that were (1) first captured and PIT tagged at one of our eight study sites located in the upper watershed (i.e., above the confluence of Big Creek and the Mainstem) and (2) genotyped for *Omy05* and genetic sex. We used recapture histories to identify “migrants”—defined as individuals that were last encountered in the lower watershed (Figure 1). Fish that were (1) detected repeatedly in the lower watershed for a period of >2 weeks (i.e., “milling”), or (2) at large for >1.5 years between initial and final encounter (i.e., “too old”) were not considered migrants.

We hypothesized that the probability of detecting a migrant (i.e., emigration probability) would vary by *Omy05* genotype and sex (Kelson et al., 2019; Pearse et al., 2019), and thus would also vary spatially in accordance with rates of gene flow and resulting *Omy05* allele frequencies. We used generalized additive models (GAMs) to estimate emigration probability as a function of length at last capture, and compared candidate models to test our hypothesis in two parts. First, to test the sex‐dependent dominance hypothesis, we used sex and genotype as categorical covariates and predicted that emigration probability would be higher for females and/or individuals with the ancestral *Omy05* inversion arrangement. Then, to quantify spatial variation in emigration probability, we considered location (site) as an additional covariate and tested the prediction that emigration probability would increase with the proportion of A alleles per site. For both predictions, we tested several models that varied in their complexity. The simplest models assumed that emigration was a function of FL, with no differences among covariates. Subsequent models considered differences among individual covariates, as well as interactions between sex and genotype (Table S2) and tributary and barrier proximity (Table S3). Goodness of fit was evaluated using Akaike Information Criterion (AIC). Spearman's rank correlation was computed to assess the relationship between emigration probability and the proportion of A alleles at each site.

#### 2.3.3 Variation in density, size structure, and sex ratio

We evaluated the relationship between *Omy05* allele frequencies and three attributes of population ecology (density, size structure, and sex ratio) using abundance and size data derived from electrofishing surveys and sex assignments determined from genetic analysis. Fish abundance at each site was estimated using the Carle and Strub (1978) method in the *FSA* package in *R* (Ogle, 2018; R Core Team, 2013). Density was calculated by dividing estimated abundance by the total wetted area (to the nearest 0.5 m^2^). Using fork length frequency distributions, we assigned all captured *O. mykiss* to one of two size/age classes: age‐0+ (i.e., <90 mm FL) and age‐1+ fish (≥90 mm FL). We estimated the density for each size class separately following the same approach as before. We tested the prediction that the density of age‐0+ fish would increase with the proportion of A alleles per site, while the density of age‐1+ fish would decrease. Females of a given size and genotype typically show a greater propensity to migrate compared to males (Pearse et al., 2019). We estimated sex ratio for each size class as the relative proportion of males and females captured at each study site, following the approach of Rundio et al. (2012). Specifically, we used exact binomial tests to evaluate whether sex ratio was equal to 50% for fish smaller than the presumed minimum threshold for outmigration/smoltification (≤120 mm FL) and male‐biased for larger fish. We used Woolf's test for homogeneity of log odds ratios to test for variation in size‐specific sex ratios among sites or years.

## 3 RESULTS

**FIGURE 2:**
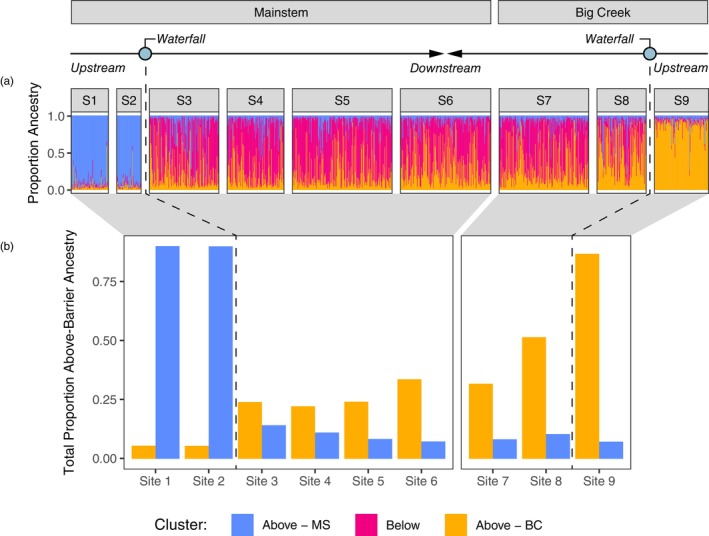
*STRUCTURE* analysis of *Oncorhynchus mykiss* assemblages across the watershed effectively divided fish into three distinct clusters, with an apparent gene flow gradient on Big Creek. Individual ancestry assignments (Q; Panel a) and total proportion ancestry to above‐barrier populations (∑Q; Panel b) depict above‐mainstem ancestry in blue, above‐Big Creek ancestry in orange, and below‐barrier ancestry in pink.

**FIGURE 3:**
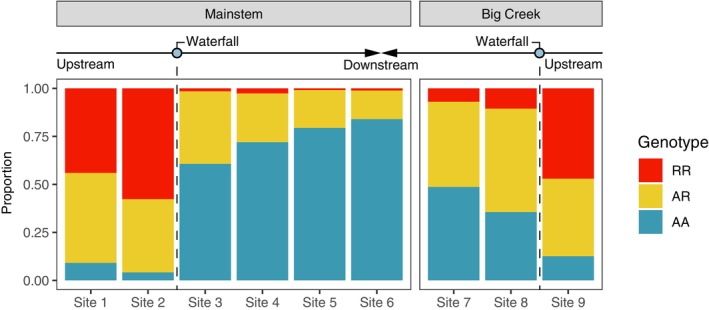
*Omy05* genotype frequencies varied across study sites as expected by selection by waterfalls and one‐way gene flow. Homozygous rearranged (RR) genotypes (red) were common above waterfalls, whereas homozygous ancestral (AA) genotypes (blue) predominated below. Below the falls, RR and AR genotypes were more prevalent at sites where we found strong signals of one‐way gene flow.

**FIGURE 4:**
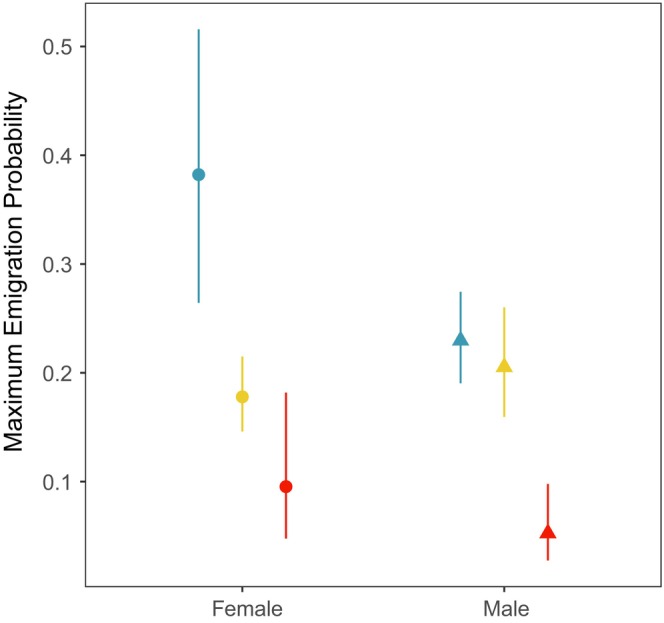
Generalized additive models (GAMs) estimated maximum emigration probability among *Omy05* genotypes and sexes in support of anadromous dominance. Female ancestral homozygous genotypes (AA, blue, left) showed a markedly elevated probability of emigrating, whereas male ancestral homozygous genotypes (AA, blue, right) showed similar emigration estimates to heterozygotes of both sexes (AR, yellow). Rearranged homozygous genotypes (RR, red) showing a reduced probability of emigrating for both sexes.

**FIGURE 5:**
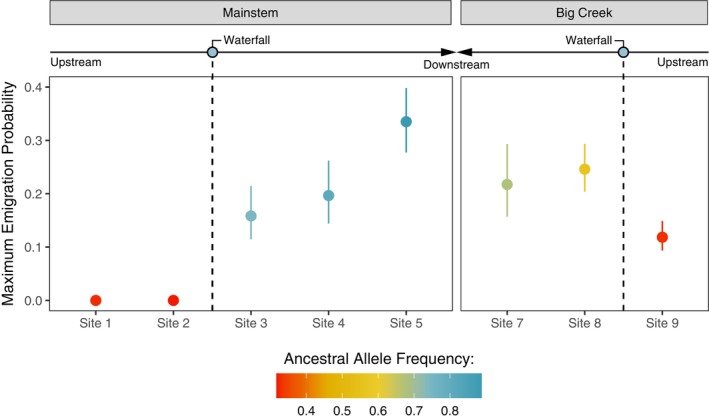
Generalized additive models (GAMs) estimated maximum emigration probability among sampling sites in accordance with observed selection and gene flow histories. For above‐falls populations, emigration probability was higher on Big Creek, supporting higher rates of dispersal and gene flow and fewer ancestral alleles at sites below.

**FIGURE 6:**
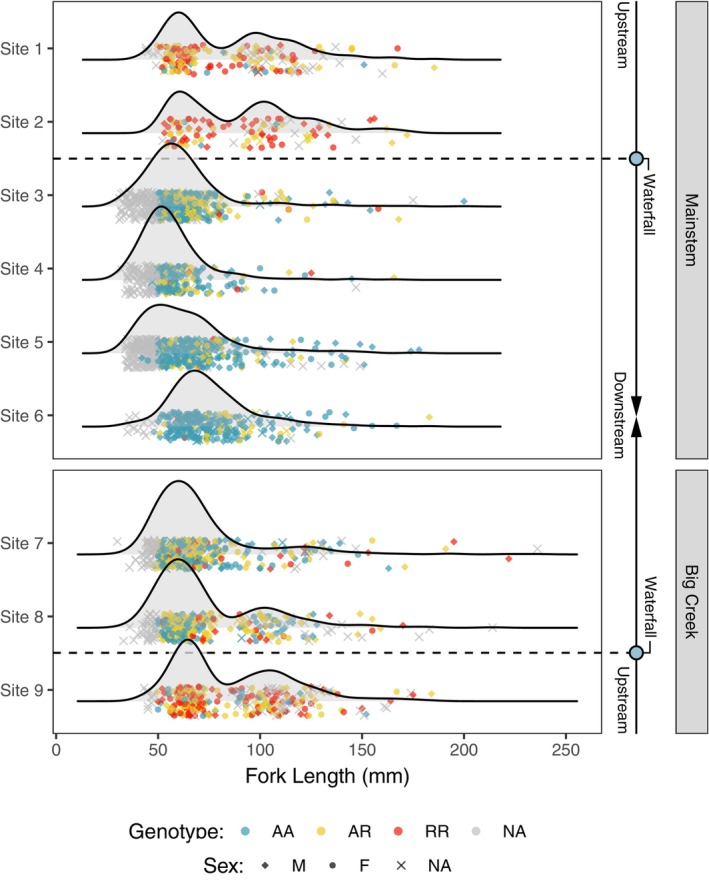
Density, size structure, and sex ratio of *O. mykiss* from each sampling site. Below‐barrier sites supported high densities of age‐0+ fish with primarily AA genotypes. Above‐barrier sites showed fewer age‐0+ fish and more age‐1+ fish with primarily RR genotypes. Below‐barrier sites on Big Creek, which received relatively high gene flow from the adjacent above‐falls population, displayed more age‐1+ fish and a greater frequency of RR genotypes compared to below‐falls sites on the mainstem, which received very low gene flow from above the falls on the mainstem.

Population‐based genetic analyses and *STRUCTURE* assignments based on neutral genetic variation revealed three distinct populations in the basin, with a strong gradient of above‐barrier ancestry below the barrier on Big Creek (Figure 2). We also found that relative frequencies of *Omy05* genotypes (Figure 3) were closely correlated with the total proportion of above‐barrier ancestry found at each study site. Generalized additive models (GAMs) confirmed that migratory behavior differs among *Omy05* genotypes, in support of anadromous dominance (Figure 4). However, emigration probability was markedly higher above the falls on Big Creek than above the falls on the Mainstem, despite similar *Omy05* genotype frequencies (Figure 5). Finally, we found that *Omy05* allele frequencies were correlated with both fish density and size structure, such that predominantly ancestral/anadromous study sites were characterized by many small fish and fewer large fish than sites with higher proportions rearranged/resident alleles (Figure 6).

### 3.1 Genomic relationships and signatures of gene flow

Genetic population structure and patterns of genetic divergence based on neutral loci were consistent with previous studies, and showed close relationships between study sites, with marginally higher *F*_ST_ values between groups separated by one or multiple barriers (Tables S4 and S5). Our population analysis included 1628 fish, representing three age‐0+ and resident cohorts which ranged from 95 to 254 fish per site. Variation in *F*_ST_ was minimal between years, so we pooled individuals from all 3 years for analyses.

*STRUCTURE* analysis provided clear cluster assignments when *k* = 3, representing three populations separated physically by waterfalls (above the Mainstem waterfall, below both barriers, and above Big Creek falls; Figure 2a). Clustering patterns were also generally consistent across alternate *k* values and across runs (Figure S3). At *k* = 2 and above, sites above the falls on the Mainstem (Site 1 and Site 2) grouped together and were differentiated from the other sites. At *k* = 3 and above, the site above the falls on Big Creek (Site 9) was also differentiated from the below‐barrier sites. At *k* = 4 and *k* = 5, some *STRUCTURE* runs roughly divided below the falls sites by tributary, but patterns were inconsistent.

Individual ancestry assignments were suggestive of genetic divergence among both above‐barrier populations, and one‐way gene flow and introgression from above to below the falls on Big Creek. Generally, most fish within each population were assigned majority ancestry to their respective cluster (Figure 2a). Likewise, the number of individuals assigned >50% above‐barrier ancestry and the total proportion ancestry to an above‐barrier cluster was higher above the falls compared to below (Figure 2b). Across all runs and populations, individual ancestries consistently included small (<5%) assignments to both outside clusters, as expected given their previously established common ancestry and relatively recent divergence. We found no evidence for a reproductively isolated group of fish below the falls represented by above‐falls migrants, as previously suggested (Pearse et al., 2009). Rather, on Big Creek many individuals below the fall contained mixed proportions of both above‐ and below‐barrier clusters, suggesting that migrants from above the falls are descending and reproducing with the below‐falls population, resulting in one‐way gene flow. Interestingly, below‐falls sites on Big Creek showed more than double the total ancestry to its above‐falls cluster (31%–52%) compared to that found on the Mainstem (7%–14%), indicative of a much higher rate of gene flow on Big Creek.

*Omy05* genotype frequencies varied considerably across study sites, and were highly consistent with patterns of divergence and one‐way gene flow found in our previous analysis (Figure 3, Table S6). At the watershed level, *Omy05* genotypes were significantly out of Hardy–Weinberg equilibrium (*D* = −80.34, *p* < 0.01), with genotype frequencies that were 51.2% homozygous ancestral (AA), 33.6% heterozygotes (AR), and 15.1% homozygous rearranged (RR) (Table S7). However, we found no evidence of Hardy–Weinberg disequilibrium in the above‐falls populations (*D*_MSA_ = 3.49, *D*_BCA_ = −3.44, both *p* > 0.25), nor in the below‐barrier tributaries (*D*_MSB_ = −0.19, *D*_BCB_ = −3.65, both *p* > 0.40) (Table S7). We found a significant positive relationship between proportion upstream ancestry and both RR genotype and R allele frequencies, leading to an increased frequency of RR genotypes and a reduced frequency of AA genotypes above both barriers. Conversely, RR genotypes were under‐represented (and AA genotypes over‐represented) below the barrier on the mainstem. However, on Big Creek below, AA genotypes were in fact fewer than what was expected and RR genotype frequencies were not significantly different from random at Big Creek below (*χ* ^2^ = 540.22, df = 6, *p* < 0.01).

### 3.2 Physical movement and migratory behavior

Our initial mark–recapture dataset included 1319 unique individuals, including 318 fish that were recaptured or detected after their initial tagging (Figure S4). A total of 184 fish met our definition of “migrants.” Of the re‐captured fish that were not migrants, the majority were re‐captured/detected at a single sampling site (*N* = 115), or at adjacent sites in a single sampling region (*N* = 16). We found only three cases where non‐migrants demonstrated more substantial intrabasin movement (i.e., relocated to more distant sites).

We found two top models exhibiting similar goodness of fit. Both models indicated ancestral/anadromous (A) dominance at *Omy05*, suggesting that the emigration probability of AR genotypes was more similar to AA genotypes than RR genotypes (Figure 4). The best‐fit model indicated A allele dominance, with differences in emigration probability between sexes, and the second‐best model represented differences only among *Omy05* genotypes (ΔAIC = 0.69). Both models outperformed the remaining alternatives, including sex‐specific dominance (Pearse et al., 2009), by a substantial margin (ΔAIC ≥6.18, Table S8). Females exhibited greater variability in their behavior and maximum emigration probability for AA females (0.38) was near double that of the other migratory genotypes (0.18–0.23, Figure 4). Maximum emigration probability for RR genotypes was substantially lower for both sexes. However, RR females demonstrated a clear peak in emigration probability (0.10) when fork length at last capture (FL_cap_) was ≥90 mm, whereas RR males demonstrated a smooth decline in emigration probability across all observed fork lengths (Figure S5).

Emigration probability varied among sampling sites in accordance with observed selection and gene flow histories (Figure 5). The best fit model of spatial variation in emigration tendency included sampling site as a covariate, where each site had a unique relationship between emigration probability and fork length (ΔAIC ≥4.27, Table S9). Emigration probability showed a weak positive correlation with the proportion of A alleles at each sampling site (ρ = 0.69, *p* = 0.07), such that peak emigration probability was higher below the falls (0.16–0.34, FL_cap_ ≥ 90 mm) than above (<0.12). However, peak emigration probability was notably higher above the falls on Big Creek (0.12) than above the falls on Scott Creek (<0.01).

### 3.3 Variation in density, size structure, and sex ratio

We captured a total of 2870 *O. mykiss* during our surveys, ranging from 33 to 225 fish per site per year, where overall abundance was largely driven by the presence of age‐0+ fish with A alleles. After accounting for habitat size, overall density ranged from 0.05 to 0.66 individuals per square‐meter among sites and years. Total fish density was highly correlated with the proportion of A alleles at a site (ρ = 0.67, *p* < 0.01, Figure S6a). The above‐barrier sites on the Mainstem (Sites 1 and 2) were consistently among the lowest density sites across years, followed by the furthest downstream site on the Mainstem (Site 6) and the above‐barrier site on Big Creek (Site 9, Figure S7). Size‐specific density estimates revealed that overall density closely reflected that of age‐0+ fish at each site (Figure S7).

We generally found higher densities of age‐1+ fish and fewer age‐0+ fish above barriers, whereas below‐barrier sites predominantly supported large densities of age‐0+ fish. Below‐barrier sites with elevated R allele frequencies size structure displayed intermediate densities of both age‐0+ and age‐1+ fish (Figure 6). Correlation tests suggested a strong positive correlation between the proportion of A alleles and density of age‐0+ fish (ρ = 0.79, *p* < 0.01, *N* = 24), and a negative correlation between the proportion of A alleles and the density of age‐1+ fish (ρ = −0.50, *p* = 0.01, *N* = 24, Figure S6b).

We found that sex ratios consistently hovered around 50% for pre‐smolt (<120 mm) fish, and became male‐biased for larger fish (Figure S8). The sex ratio for pre‐smolt fish in the basin did not differ from 50% (95% CI = 0.49–0.58, *p* = 0.16, *N* = 521). However, sex ratio for large fish (>120 mm) was male‐biased (95% CI = 0.57–1.00, *p* < 0.01, *N* = 131). The relationship between size and sex was consistent among sites (*χ* ^2^ = 12.26, df = 8, *p* = 0.14) and years (*χ* ^2^ = 4.70, df = 2, *p* = 0.10).

## 4 DISCUSSION

Our study examined how gene flow from resident *O. mykiss* populations located above barriers shapes genetic, phenotypic, and population variation in the downstream anadromous populations. We found genetic evidence demonstrating downstream dispersal from resident to anadromous populations (Q1) on one of two tributaries (Big Creek), and that above‐barrier dispersers reproduce with below‐barrier fish to facilitate one‐way gene flow. The contrasting patterns of gene flow between tributaries provided an ideal setting for assessing the eco‐evolutionary consequences of gene flow for the downstream populations. We found higher frequencies of R alleles/RR genotypes at below‐barrier study sites where gene flow was apparent. We identified phenotypic evidence of downstream dispersal from resident to anadromous populations (Q2) based on variation in migratory tendency among individuals and study sites, including an increased migration probability for the above barrier population on Big Creek. Finally, we demonstrate that one‐way gene flow impacts the density and size structure of populations (Q3) through shifts in life‐history strategy. These results inform our overall understanding of the eco‐evolutionary consequences of one‐way gene flow, with implications for conservation and management.

### 4.1 Genomic relationships and signatures of gene flow

Patterns of genetic divergence and shared ancestry indicated that individuals from one of two resident populations have descended the falls and introgress with the downstream population. Despite representing relatively recently introduced populations, cluster assignments consistently indicated that above‐barrier populations are genetically differentiated from downstream (below‐falls) populations. However, a substantial signature of gene flow, as evidenced by above‐barrier ancestry in below‐barrier individuals, was apparent only on Big Creek. As such, rates of dispersal and gene flow may be context‐specific. This contrasting pattern provides an opportunity to explore potential eco‐evolutionary outcomes by comparing the tributary with gene flow (Big Creek) to that without gene flow (Mainstem). Although a formal pre‐introduction temporal control was not possible given the historic nature of this introduction, previous records suggest that the below‐barrier population in Scott Creek was almost entirely anadromous (phenotypically) and that the founders of the above‐barrier populations originated from transplanting those anadromous fish upstream (Hayes et al., 2004; Pearse et al., 2014; Shapovalov & Taft, 1954). Moreover, many studies have shown that in the absence of migratory barriers, similar coastal watersheds are consistently dominated by anadromous phenotypes/ancestral *Omy05* alleles (e.g. Apgar et al., 2017). Thus, the genotype frequencies of the population of fish introduced above both barriers would have likely resembled our downstream anadromous study sites.

Interestingly, the signal of neutral variation and presence of mixed‐ancestry individuals on Big Creek indicate that some level of interbreeding occurs between dispersing residents from above the falls and anadromous fish immediately below the falls. Previous studies have suggested that fish that descend from over the falls remain largely reproductively isolated from their anadromous counterparts (Pearse et al., 2009). However, STRUCTURE assignments clearly assigned mixed ancestry to individuals in the below‐barrier population, and signals of introgression extended further downstream than previous records. Although some degree of reproductive isolation may occur, it is evident that above‐barrier fish do successfully reproduce with anadromous fish below barriers.

We found that one‐way dispersal from resident‐adapted populations above the falls can influence the frequency of *Omy05* genotypes in anadromous populations downstream. Thus, both fragmentation by migratory barriers and the rate of subsequent gene flow can drive life‐history evolution within a population. *Omy05* genotype patterns matched our prediction that R alleles/RR genotypes would be more common above barriers, where strong selection against migration occurs. *Omy05* genotype frequencies were strongly associated with downstream gene flow, as represented by the positive relationship R alleles/RR genotypes and the proportion of upstream ancestry found at a given site. Most notably, we found relatively high proportions of R alleles/RR genotypes at historically anadromous sites on Big Creek, where we also found a strong signature of above‐barrier gene flow.

### 4.2 Physical movement and migratory behavior

We found that *Omy05* genotype and sex both influence the probability of an individual emigrating from the upper watershed, providing additional support for genetic control of migratory behavior at an individual level (Kelson et al., 2019; Pearse et al., 2019). Both of our top models supported an ancestral/anadromous (A) dominant basis for migration, and one model indicated differences in emigration probability among males and females with the same genotype. This result is contrary to the sex dominance hypothesis proposed by Pearse et al. (2019), which posits that the ancestral (A) allele is dominant in females (i.e., female heterozygotes more likely to migrate), whereas the rearranged (R) allele is dominant in males (i.e., male heterozygotes more likely to remain resident). Although we found an overall greater probability of migration in AA females, we did not find a pattern of sex dominance. Furthermore, differences in maximum emigration probability were negligible among males and females of the other genotypes. These observations add to a growing number of cases demonstrating that the individual‐level relationship between sex, *Omy05* genotype, and migration can vary across watersheds (Kelson et al., 2019; Pearse et al., 2019).

Given the strong correlation between *Omy05* genotype and migration, we expected emigration probability to correlate with genotype frequencies at the site level. Our observations generally supported this prediction. Above‐barrier populations were less likely to outmigrate than those below. However, we also found that fish from above the falls on Big Creek were more likely to descend the falls and outmigrate compared to those found above the Mainstem waterfall, despite similar frequencies of A alleles. While this observation reinforces our conclusions that gene flow from above the falls is more common on Big Creek than on the Mainstem, it also indicates that migratory behavior can be context dependent. Previous research has proposed a number of physiological and environmental factors that may influence migration decisions in *O. mykiss*, including temperature, density, and individual growth rate (reviewed in Kendall et al., 2015). Moreover, recent research has suggested that physiological mechanisms such as growth could mediate the relationship between *Omy05* and migratory behavior, facilitating indirect and environmentally dependent genetic control of migration (Kelson, Miller, et al., 2020). One possibility is that higher overall densities above the falls on Big Creek compared to the Mainstem contribute to a higher rate of downstream dispersal (Figure S7). We suggest that future studies explore further how environmental variables, juvenile physiology, and *Omy05* genotypes interact to influence migration behavior.

### 4.3 Variation in density, size structure, and sex ratio

The size structure of fish populations closely corresponds to patterns of adaptation and dispersal revealed by our genetic and phenotypic analyses. We found a positive relationship between overall density and A allele frequency, primarily driven by higher densities of age‐0+ individuals. This pattern is consistent with the fact that, compared to resident conspecifics, anadromous females are substantially larger and more fecund. Conversely, we observed lower densities of small fish and higher densities of large fish at sites with predominantly R alleles, representative of larger, older fish that do not emigrate. We found intermediate densities of both small and large fish found below the barrier on Big Creek, but not on the Mainstem, suggesting that dispersal from resident‐adapted populations above barriers can clearly impact the density and size structure of historically anadromous populations below; this is the signature of dispersal and gene flow.

We show that large fish exhibited male‐biased sex ratios consistently among sites and years, with no apparent relationship to population or gene flow. This pattern complements previous research in Scott Creek that has shown that anadromous returns are female‐biased (Hayes et al., 2004), and supports the overall notion that the costs and benefits of migration are often asymmetrical among sexes in salmonids and results in differential rates of anadromy between sexes (Fleming & Reynolds, 2004; Hendry et al., 2004).

If gene flow from a divergent population shapes variation in the population density, size structure, and sex ratio of its founding population, it can, by extension, have significant impacts on virtually every level of ecological organization. The ecological consequences of variation in population demographics have been extensively studied in salmonid populations, in particular. For example, variation in population density can have profound effects on juvenile growth and survival, and substrate/nutrient transport (Essington et al., 2000; Gende et al., 2002; Imre et al., 2005; Moore & Schindler, 2008). The evolution of salmonid body size has been shown to directly impact fecundity, nest size, food web subsidies, and nutrient cycling (Carlson et al., 2011; Oke et al., 2020; Steen & Quinn, 1999). And skewed sex ratios can directly alter reproductive competition and mating behavior, and elicit indirect ecological consequences via sexual dimorphism (Holtby & Healey, 1990; Kendall & Quinn, 2013; Quinn et al., 2010; Quinn & Foote, 1994). While the aforementioned examples are not an exhaustive list, they serve to illustrate the point that gene flow can shape a wide range of ecological processes, extending the ecological consequences of rapid adaptive evolution beyond the population in which it evolved. This perspective adds to a growing literature documenting the importance of evolutionary processes in shaping ecological outcomes, and highlights the potential for the *Omy05* inversion to act as a keystone gene.

### 4.4 Management implications

The effects of migratory barriers, habitat permeability, and population fragmentation on population structure and dynamics are common concerns across a wide variety of protected and managed taxa (Alcaide et al., 2009; Frankham et al., 2017; Peterman et al., 2014; Van Moorter et al., 2020). In this case, anadromous steelhead populations are listed as endangered or threatened in a large portion of their native range under the US Endangered Species Act (National Marine Fisheries Service, 2016). However, ESA listings currently exclude resident populations from protection, even when they are located within the same watershed and may be influencing the evolution and ecology of downstream anadromous populations through gene flow.

Our results add to a growing conversation regarding the role of genomic diversity in the conservation and recovery of species and ecosystems (Oke & Hendry, 2019; Stange et al., 2021; Waples et al., 2022; Waples & Lindley, 2018). We demonstrate that upstream resident populations indeed disperse downstream and impact anadromous populations at a genetic, phenotypic, and population level. Importantly, gene flow from resident fish appears to play a role in maintaining life history diversity in predominantly anadromous populations, which may support long‐term population resilience (Apgar et al., 2017; Schindler et al., 2010). These dynamics are also important from an ecosystem‐based management standpoint because population interactions are likely shaping ecological outcomes for communities and ecosystems. In order to implement effective conservation and management interventions for species and ecosystems, there is a critical need for understanding how locally adapted populations exchange genes and thus influence one another from an eco‐evolutionary perspective.

## 5 CONCLUSIONS

We conclude that gene flow can extend the ecological consequences of rapid adaptive evolution beyond the population in which it evolved, adding to a growing literature documenting the importance of evolutionary processes in shaping ecological outcomes. We show that one‐way gene flow can influence the distribution of key functional variation at both a genetic and phenotypic level. Furthermore, we demonstrate that the rapid evolution of life‐history strategy can alter the ecological structure of populations. This chain of influence from genes to phenotypes to populations has a strong potential to extend to community structure and ecosystem function (i.e., a keystone gene framework). Finally, we recommend that management and conservation efforts carefully assess the potential for gene flow to influence ecological outcomes.

## CONFLICT OF INTEREST STATEMENT

The authors declare no conflicts of interest.

## Supporting information


Appendix S1.

## Data Availability

The data that support the findings of this study are openly available at https://doi.org/10.5061/dryad.cvdncjtbh
