# Supplementary material for: Genetic divergence and one‐way gene flow influence contemporary evolution and ecology of a partially migratory fish
Source: Evol Appl. 2024 Jun 21;17(6):e13712. doi: 10.1111/eva.13712 (PMC11192968; doi:10.1111/eva.13712)
Supplement: Supplementary file 1 — Appendix S1. [file EVA-17-e13712-s001.pdf]

Genetic divergence and one-way gene flow influence contemporary evolution and ecology of a partially migratory fish

Supplementary Material

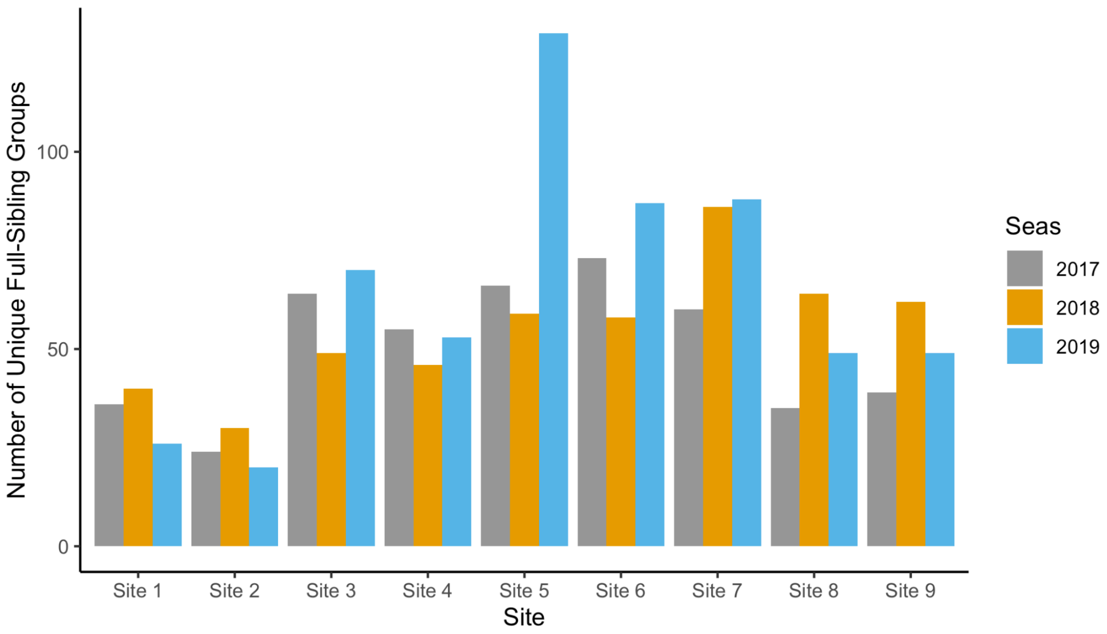

Figure S1. Number of unique full-sibling groups found at each study site across years. The number of families per site ranged from 20 to 130 unique full sibling groups. Families that were spread across multiple sites and/or years were seldom, and primarily consisted of siblings spread across adjacent sampling sites within a study year, consistent with patterns expected by juvenile dispersal during higher flows.

	Family Size Frequency																			
	Number of Siblings in Family																			
	**1**	**2**	**3**	**4**	**5**	**6**	**7**	**8**	**9**	**10**	**11**	**12**	**13**	**14**	**15**	**16**	**17**	**18**	**19**	**20**
**Number of Observations**	1212	140	23	25	18	10	10	12	9	6	3	5	5	2	1	2	2	1	1	1
**Proportion of Total**	0.815	0.094	0.015	0.017	0.012	0.007	0.007	0.008	0.006	0.004	0.002	0.003	0.003	0.001	0.001	0.001	0.001	0.001	0.001	0.001

Table S1. Number of observations of full-sibling groups of a given size. Most families were singletons or pairs (~90%). Families consisted mostly of singletons and pairs, representing 81% and 9% of all families respectively. The largest family included 20 full siblings.

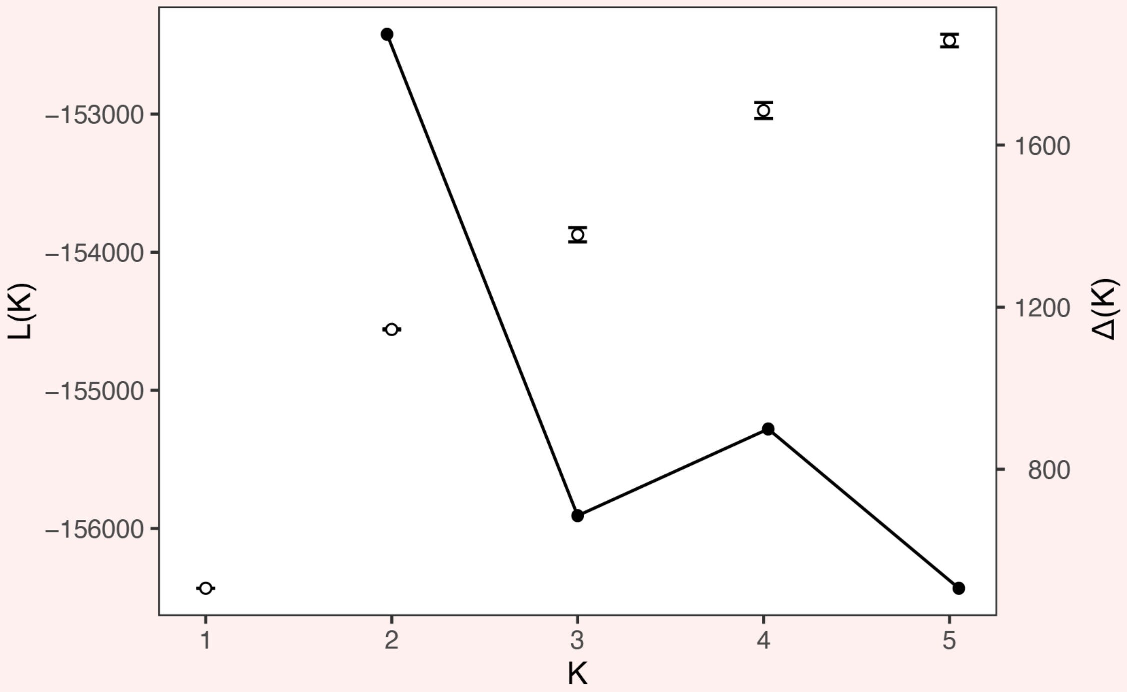

Figure S2. Bayesian inference for number of clusters (K) among subpopulations. We focused our STRUCTURE analysis on a K value that maximized the structure present in the data while still considered biologically reasonable to test our hypothesis, following the methods of Liliana Porras-Hurtado et al. (2013). Thus, we chose to focus on K=3, which we believe to be the most biologically relevant value for K given the physical landscape and natural history of our study system. This value was further validated by considering: (1) mean log probability (L(K) ±SD; white; Pritchard et al. 2000); and (2) the second order rate of change of the likelihood function with respect to K (ΔK; black; Evanno et al. 2005).

		Proposed Models					
					Sex-Genotype Interractions		
		Fork Length Only[7]	Sex-Only Differences[2]	Genotype-Only Differences[3]	Residence Dominance[4]	Anadromous Dominance[5]	Sex-Dependent Dominance[6]
Female	AA	A	A	A	A	A	A
	AR	A	A	B	B	A	A
	RR	A	A	C	B	B	B
Male	AA	A	B	A	C	C	C
	AR	A	B	B	D	C	D
	RR	A	B	C	D	D	D

[1] Assumes emigration probability is a smooth function of fork length, with no variation across sex or genotype

[2] Males and females differ in their emigration probability, independently of genotype

[3] Genotypes differ in their emigration probability, independently of sex

[4] Heterozygotes (AR) behave similarly to rearranged homozygotes (RR) with sex differences

[5] Heterozygotes (AR) behave similarly to ancestral homozygotes (AA) with sex differences

[6] Heterozygote females (FAR) behave similarly to ancestral homozygotes (FAA) whereas heterozygote males (MAR) behave similarly to rearranged hymozygotes (MRR)

[7] Unique smooth for each sex and genotype combination

Table S2. GAM covariates representing alternative sex/genotype interactions. All GAMs modeled emigration probability as a function of fork length at most recent capture, with alternative covariate groupings representing hypothesized relationships between sex/genotype and life history expression.

			Proposed Models							
						Spatial Variable Interractions				
			Fork Length Only[1]	Above vs. Below[2]	Tributary Differences[3]	Residency Above[4]	Anadromy Below[5]	Big Creek Gene Flow[6]	Scott Creek Gene Flow[7]	Subpopulati Differences
Mainstem	Above	Site 1	A	A	B	A	C	B	C	C
	Above	Site 2	A	A	B	A	C	B	C	C
	Below	Site 3	A	B	B	C	B	C	C	D
	Below	Site 4	A	B	B	C	B	C	C	D
	Below	Site 5	A	B	B	C	B	C	C	D
	Below	Site 6	A	B	B	C	B	C	C	D
Big Creek	Below	Site 7	A	B	A	B	B	A	B	B
	Below	Site 8	A	B	A	B	B	A	B	B
	Above	Site 9	A	A	A	A	A	A	A	A

[1] Assumes emigration probability is a smooth function of fork length, with no variation across space
[2] Above and below subpopulations demonstrate alternative migratory behaviors
[3] Migratory behavior differs among tributaries, with no effect of barriers
[4] Above barrier subpopulations demonstrate similar migratory behavior, below barrier subpopulations vary
[5] Below barrier subpopulations demonstrate similar migratory behavior, above barrier subpopulations vary
[6] Big Creek sub-populations demonstrate similar migratory behavior, Scott Creek subpopulations vary
[7] Scott Creek sub-populations demonstrate similar migratory behavior, Big Creek subpopulations vary
[8] Unique smooth for each sub-population
[9] Unique smooth for each site

Table S3. GAM covariates representing alternative spatial patterns. All GAMs modeled emigration probability as a function of fork length at most recent capture, with alternative covariate groupings representing hypothesized relationships between geographic location and life history expression.

Tributary	Population	Sample size	Loci typed	Unbiased Hz	Unbiased Hz SD	Obs Hz	Obs Hz SD	No Alleles	Alleles SD
Mainstem	Site 1	95	92	0.3508	0.0148	0.3409	0.0051	1.93	0.25
	Site 2	69	92	0.3567	0.0151	0.3546	0.0060	1.95	0.23
	Site 3	171	92	0.3815	0.0121	0.3782	0.0039	2.00	0.00
	Site 4	128	92	0.3800	0.0120	0.3781	0.0045	2.00	0.00
	Site 5	254	92	0.3820	0.0117	0.3784	0.0032	2.00	0.00
	Site 6	218	92	0.3887	0.0115	0.3896	0.0034	2.00	0.00
Big Creek	Site 7	230	92	0.3938	0.0113	0.3922	0.0034	2.00	0.00
	Site 8	117	92	0.3938	0.0120	0.3952	0.0047	2.00	0.00
	Site 9	138	92	0.3675	0.0143	0.3730	0.0043	1.97	0.18

Table S4. Summary statistics for subpopulation sampling. Sample sizes ranged from 69 to 254 individuals per sample site, and we retained 92 loci that met our filtering criteria. Above-barrier subpopulations (gray) had marginally reduced alleles per locus (~1.95) compared to below-barrier subpopulations (white), and a similar pattern was observed for heterozygosity.

Pairwise F_{ST} - All Years

			Mainstem						Big Creek		
			Above		Below				Below		Above
			Site 1	Site 2	Site 3	Site 4	Site 5	Site 6	Site 7	Site 8	Site 9
Mainstem	Above	Site 1	0								
		Site 2	0.01626	0							
	Below	Site 3	0.07331	0.07413	0						
		Site 4	0.07288	0.07472	0.003428	0					
		Site 5	0.08044	0.08027	0.006013	0.002036	0				
		Site 6	0.08205	0.07943	0.01035	0.006996	0.006957	0			
Big Creek	Below	Site 7	0.07547	0.07581	0.0115	0.0093	0.008411	0.003821	0		
		Site 8	0.07455	0.07512	0.01654	0.0176	0.01472	0.0106	0.005526	0	
	Above	Site 9	0.08241	0.08448	0.03924	0.04179	0.03898	0.03587	0.03678	0.02426	0

Table S5. Pairwise F_{ST} values. Patterns of genetic divergence based on neutral loci were consistent with previous studies. Pairwise F_{ST} showed close relationships between study sites with marginally higher F_{ST} values between groups separated by one or multiple barriers

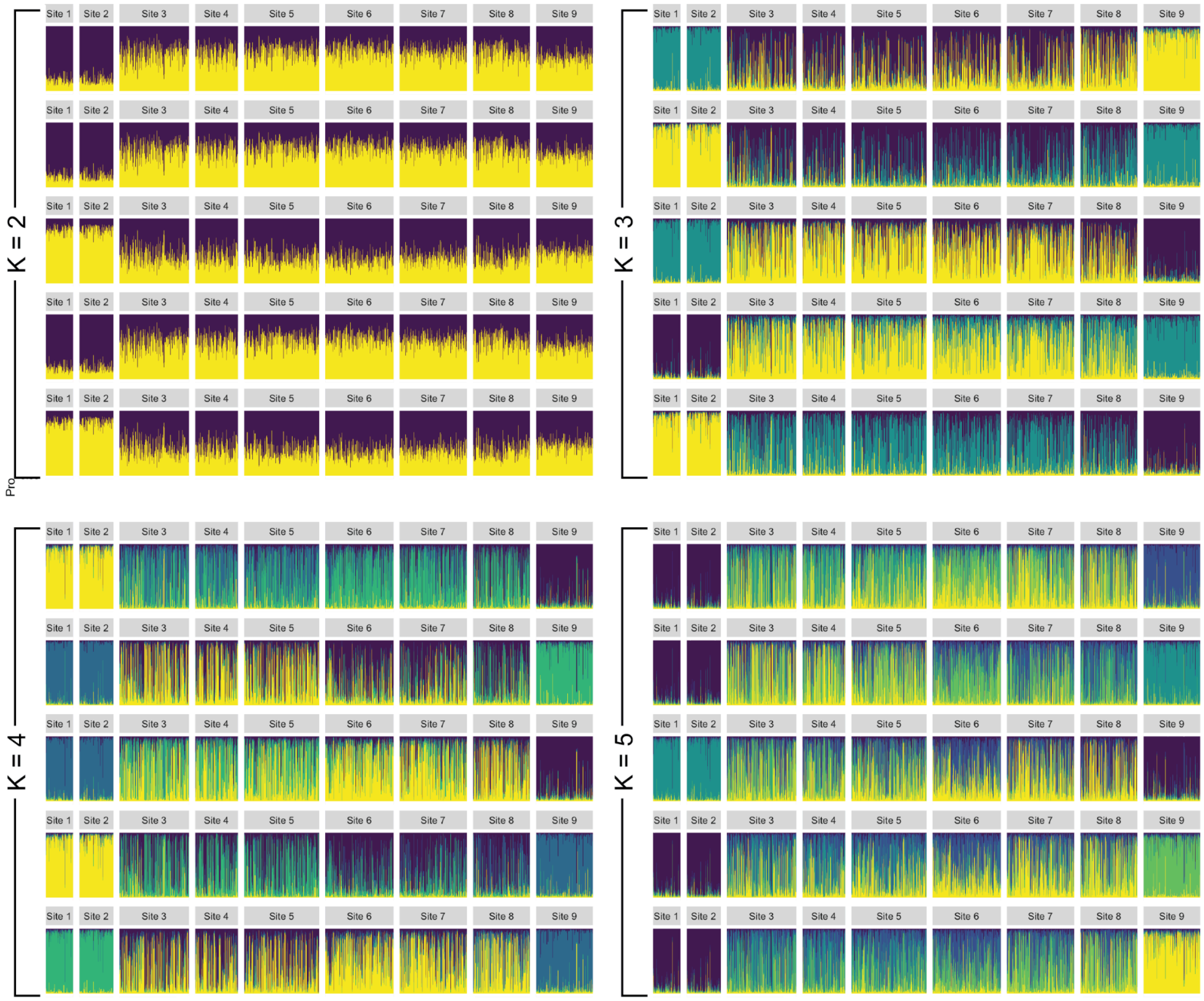

Figure S3. STRUCTURE Results. Model-based clustering analysis of *O. mykiss* captured at one of nine sampling sites in the Scott Creek watershed using STRUCTURE at k = 2–5. Each line represents an individual, and the proportion of color indicates the posterior probability of ancestry to a specific cluster. Above barrier subpopulations on the mainstem (Sites 1-2) and Big Creek (Site 9) were consistently differentiated from fish below the barrier, with signals of mixed above-below ancestry on Big Creek.

			N			Proportion		
**Tributary**	**Proximity**	**Site**	**AA**	**AR**	**RR**	**AA**	**AR**	**RR**
Mainstem	Above	Site 1	13	67	63	0.09	0.47	0.44
		Site 2	4	37	56	0.04	0.38	0.58
	Below	Site 3	199	124	5	0.61	0.38	0.02
		Site 4	136	48	5	0.72	0.25	0.03
		Site 5	258	64	3	0.79	0.20	0.01
		Site 6	220	39	3	0.84	0.15	0.01
Big Creek	Below	Site 7	181	165	26	0.49	0.44	0.07
		Site 8	101	153	30	0.36	0.54	0.11
	Above	Site 9	32	103	120	0.13	0.40	0.47

Table S6. Summary of genotype frequencies at each sampling site. *Omy05* genotype frequencies varied considerably across survey sites. Homozygous rearranged (RR) genotypes were common above waterfalls, whereas homozygous ancestral (AA) genotypes predominated below. Below the falls, RR and AR genotypes were more prevalent at sites where we found strong signals of one-way gene flow.

Test Group	D	p-value
**Watershed ***	**-80.34133**	**1.55E-19**
Cluster 1 (Mainstem Above)	3.491745	0.3421181
Site 1	2.444737	0.3549063
Site 2	1.076923	0.8280023
**Cluster 2 (Below) ***	**-14.65444**	**0.0109681**
Mainstem Below	-0.189781	0.8847993
Site 3	2.163288	0.5481742
Site 4	0.6113281	1
Site 5	0.5600394	1
Site 6	-1.255734	0.389153
Big Creek Below	-3.65679	0.4372264
Site 7	2.088043	0.623661
Site 8	-3.964286	0.2155192
Cluster 3 (Big Creek Above)	-3.437166	0.2571284
Site 9	-3.437166	0.2571284

Table S7. Exact tests for Hardy-Weinberg equilibrium. *Omy05* genotypes were significantly out of Hardy-Weinberg equilibrium at the watershed level ($D = -80.34$, $P < 0.01$), and in the below-falls subpopulation ($D = -14.65$, $P < 0.05$). However, we found no evidence of Hardy-Weinberg disequilibrium in the above-falls subpopulations, within either below-barrier tributary, or at the site level.

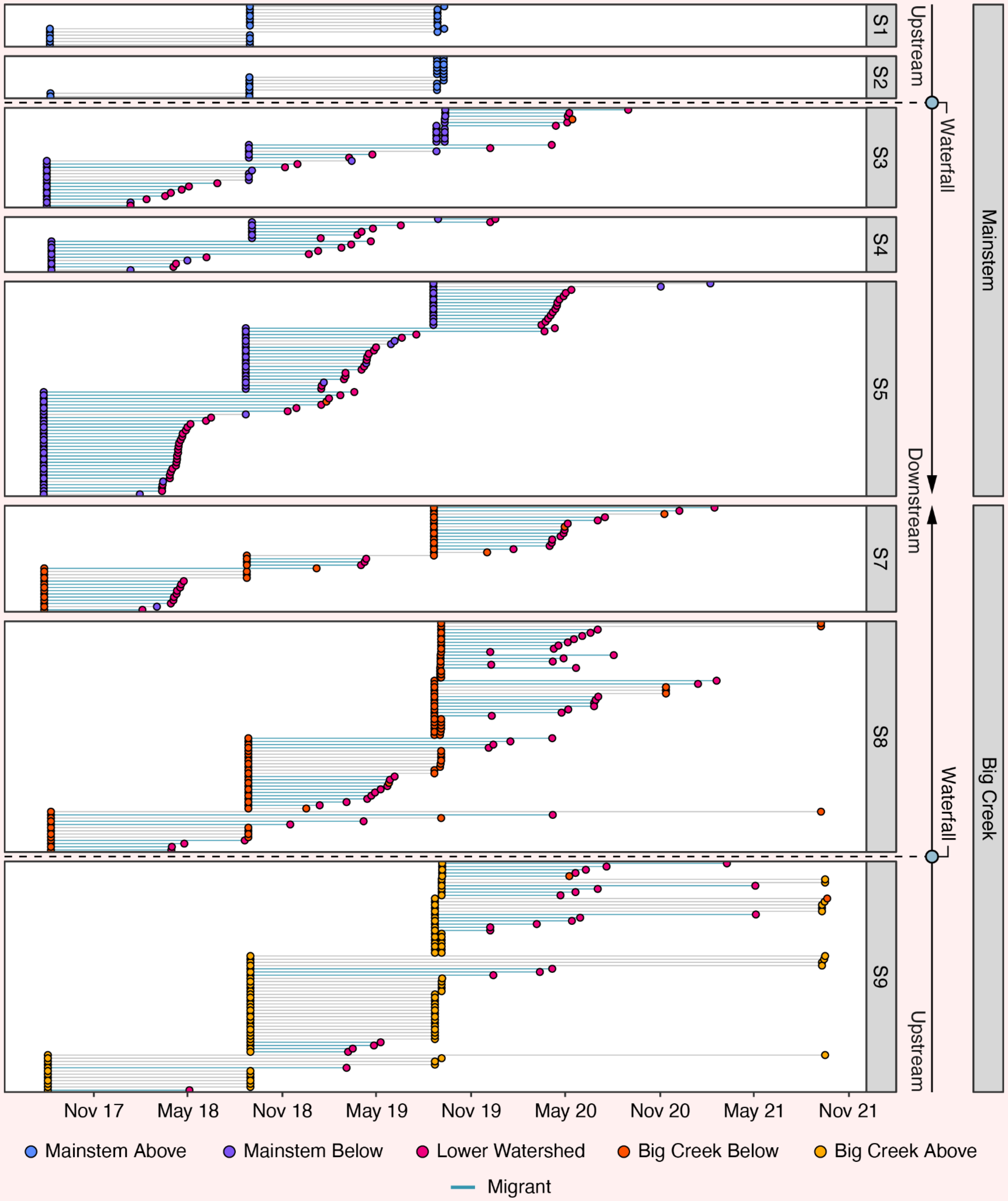

Figure S4. Individual Observation Histories. Recapture/detection histories for individual fish that were observed more than once during the study. Each line represents an individual fish, with observation events (circles) colored by encounter location. Individuals are arranged according to the location (census site) of their initial encounter.

Migration by Sex and Genotype

Model Rankings

Rank	Model	Formula	aic	Δaic
**1**	**Anadromous Dominance**	`detect ~ s(fl, by = anadsex) + anadsex`	1015.135	0.0000000
2	Genotype Effect	`detect ~ s(fl, by = geno) + geno`	1015.825	0.6896946
3	Full Interaction	`detect ~ s(fl, by = unspec) + unspec`	1021.311	6.1760612
4	Sex-Dependent Dominance	`detect ~ s(fl, by = sexdep) + sexdep`	1029.754	14.6195042
5	Residence Dominance	`detect ~ s(fl, by = ressex) + ressex`	1051.513	36.3785382
6	Fork Length	`detect ~ s(fl)`	1058.418	43.2834791
7	Sex Effect	`detect ~ s(fl, by = sex)`	1058.950	43.8154244

Table S8. Model selection rankings for sex/genotype GAMs. Both top models indicated ancestral/anadromous (A) dominance at Omy05, and the top-ranked model also indicated differences in emigration probability between sexes. Both models outperformed the remaining alternatives by a substantial margin (ΔAIC ≥ 6.18, Table S7).

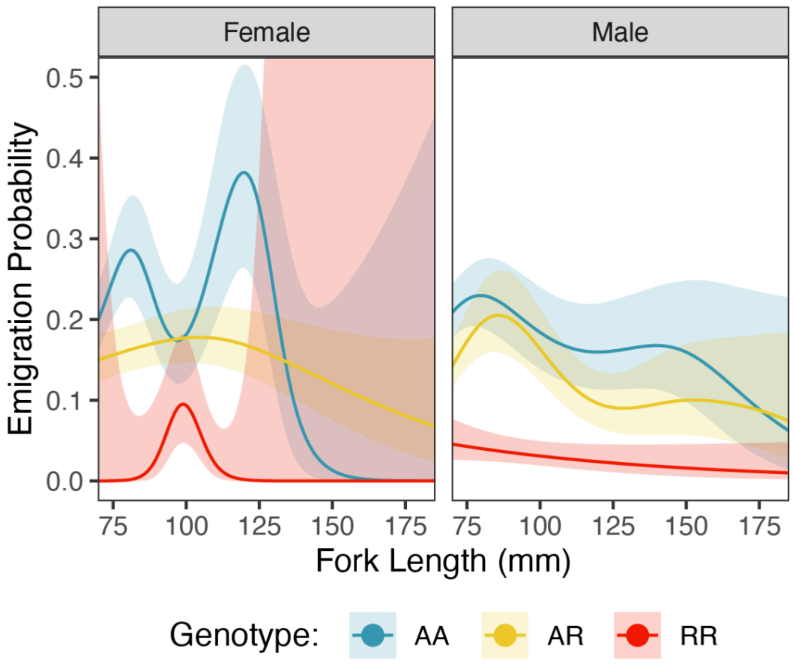

Figure S5. Model predictions for sex/genotype GAMs. Full model predictions for probability of emigration as a function of fork length at most recent capture, as estimated by GAMs using genotype and sex as separate categorical covariates.

Spatial Variation in Migration				
Model Rankings				
Rank	Model	Formula	aic	Δaic
**1**	**Site Differences**	`detect ~ s(fl, by = h6) + h6`	971.6307	0.000000
2	Subpopulation Differences	`detect ~ s(fl, by = h5) + h5`	975.8957	4.265067
3	Big Creek Gene Flow	`detect ~ s(fl, by = h3) + h3`	979.0051	7.374419
4	Anadromy Below	`detect ~ s(fl, by = h2) + h2`	980.5094	8.878797
5	Residency Above	`detect ~ s(fl, by = h1) + h1`	1004.0966	32.465906
6	Barrier Proximity	`detect ~ s(fl, by = prox) + prox`	1008.7103	37.079635
7	Scott Creek Gene Flow	`detect ~ s(fl, by = h4) + h4`	1042.6874	71.056787
8	Tributary	`detect ~ s(fl, by = trib) + trib`	1045.7968	74.166139
9	Base Model	`detect ~ s(fl)`	1058.4183	86.787637

Table S9. Model selection rankings for spatial pattern GAMs. The best fit model included sampling site as a covariate, where each site had a unique relationship between emigration probability and fork length (ΔAIC ≥ 4.27, Table S9).

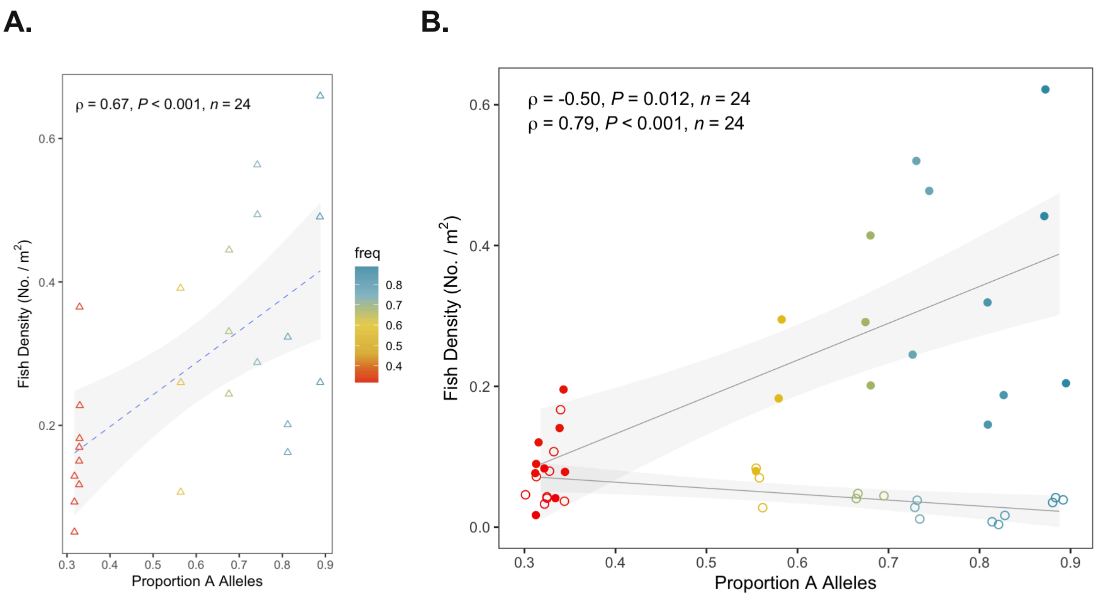

Figure S6. Site Density Estimates and *Omy05* Allele Frequencies. (A) Total fish density was highly correlated with the proportion of anadromous alleles at a site ($\varrho = 0.67$, $P < 0.01$). (B) Density of small fish (filled points) showed a positive correlation with anadromous allele frequency ($\varrho = 0.79$, $P < 0.01$), whereas the density of large fish (empty points) showed a negative correlation with anadromous allele frequency ($\varrho = -0.50$, $P < 0.05$).

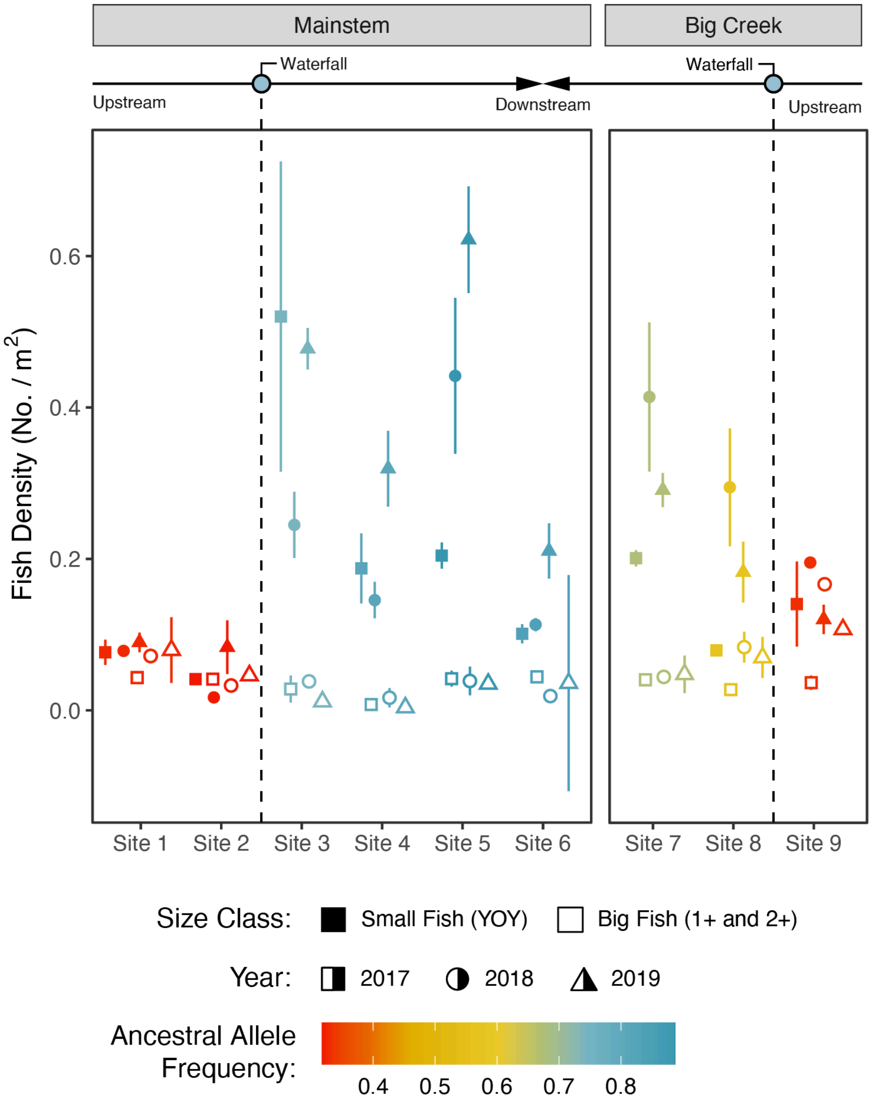

Figure S7. Site Density Estimates by Size Class. Fish density for small fish (filled points) and big fish (empty points) at each sampling site. Shape represents sampling year, and color represents relative frequency of ancestral (A) Omy05 alleles. Above-falls sites (Sites 1-2, 9) had low frequencies of A alleles, low densities of small fish, and increased densities of large fish relative to below-falls sites. However, below-falls sites on Big Creek (Sites 7-8) showed intermediate frequencies of A alleles and increased densities of large fish relative to the mainstem-below sites (Sites 3-6).

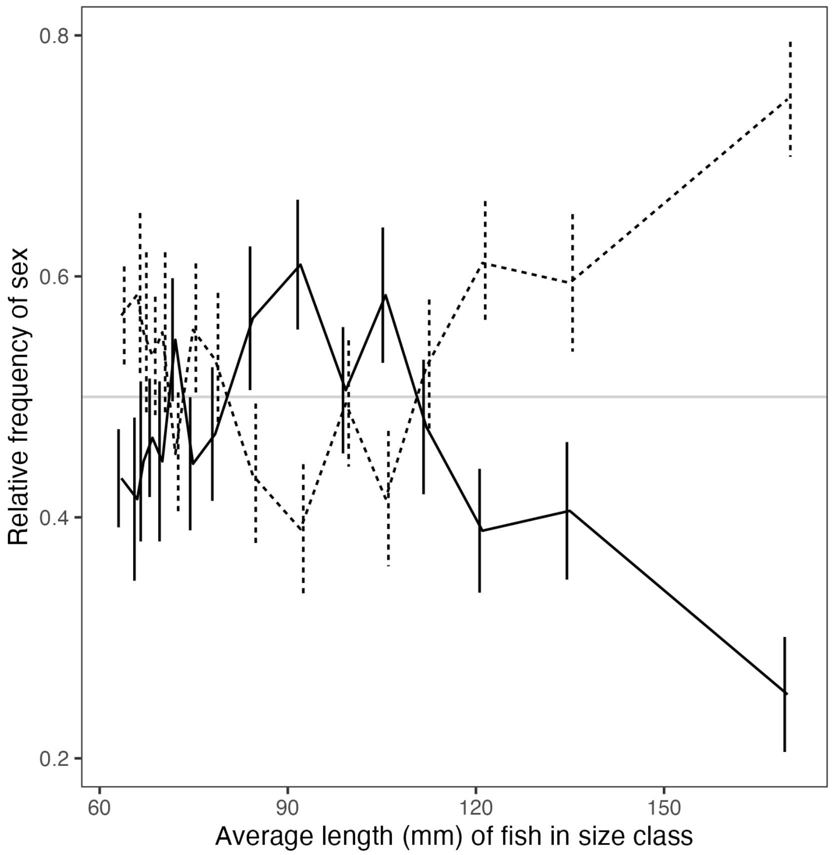

Figure S8. Size-Specific Sex Ratios. Relative proportion of males (dashed) and females (solid) by size class. The sex ratio for pre-smolt fish (≤ 120 mm) fish in the basin did not differ from 50% (95% CI = 0.49 - 0.58, p = 0.16, n = 521). However, sex ratio for large fish (> 120 mm) was male-biased (95% CI = 0.57 - 1.00, P < 0.01, n = 131). The relationship between size and sex was consistent among sites (□^{2} = 12.26, df = 8, P = 0.14) and years (□^{2} = 4.70, df = 2, P = 0.10).
